# Cortical Thickness, Surface Area and Subcortical Volume Differentially Contribute to Cognitive Heterogeneity in Parkinson’s Disease

**DOI:** 10.1371/journal.pone.0148852

**Published:** 2016-02-26

**Authors:** Niels J. H. M. Gerrits, Anita C. van Loenhoud, Stan F. van den Berg, Henk W. Berendse, Elisabeth M. J. Foncke, Martin Klein, Diederick Stoffers, Ysbrand D. van der Werf, Odile A. van den Heuvel

**Affiliations:** 1 Department of Anatomy & Neurosciences, VU University medical center (VUmc), Amsterdam, the Netherlands; 2 Department of Neurology, VUmc, Amsterdam, the Netherlands; 3 Netherlands Institute for Neuroscience, an Institute of the Royal Netherlands Academy of Arts and Sciences, Amsterdam, the Netherlands; 4 Department of Medical Psychology, VUmc, Amsterdam; 5 Department of Psychiatry, VUmc, Amsterdam, the Netherlands; 6 Neuroscience Campus Amsterdam (NCA), Amsterdam, the Netherlands; Chinese Academy of Sciences, CHINA

## Abstract

Parkinson’s disease (PD) is often associated with cognitive deficits, although their severity varies considerably between patients. Recently, we used voxel-based morphometry (VBM) to show that individual differences in gray matter (GM) volume relate to cognitive heterogeneity in PD. VBM does, however, not differentiate between cortical thickness (CTh) and surface area (SA), which might be independently affected in PD. We therefore re-analyzed our cohort using the surface-based method FreeSurfer, and investigated (i) CTh, SA, and (sub)cortical GM volume differences between 93 PD patients and 45 matched controls, and (ii) the relation between these structural measures and cognitive performance on six neuropsychological tasks within the PD group. We found cortical thinning in PD patients in the left pericalcarine gyrus, extending to cuneus, precuneus and lingual areas and left inferior parietal cortex, bilateral rostral middle frontal cortex, and right cuneus, and increased cortical surface area in the left pars triangularis. Within the PD group, we found negative correlations between (i) CTh of occipital areas and performance on a verbal memory task, (ii) SA and volume of the frontal cortex and visuospatial memory performance, and, (iii) volume of the right thalamus and scores on two verbal fluency tasks. Our primary findings illustrate that i) CTh and SA are differentially affected in PD, and ii) VBM and FreeSurfer yield non-overlapping results in an identical dataset. We argue that this discrepancy is due to technical differences and the subtlety of the PD-related structural changes.

## Introduction

In addition to typical motor symptoms such as tremor, bradykinesia, rigidity, and postural instability, patients with Parkinson’s disease (PD) often experience non-motor symptoms. Among these non-motor symptoms are cognitive deficits, which predominantly exist in the domain of executive functions, memory and visuospatial performance [1, 2]. Cognitive deficits are common, even in early stage PD [2], and up to 80% of all patients suffer from dementia at the end-stage of the disease [3]. The onset and rate of cognitive decline, however, differs considerably between patients.

Recently, we showed that differences in brain structure may contribute to cognitive heterogeneity in PD [4]. In that VBM study patients had relatively small areas of decreased gray matter (GM) volume in cortical areas such as the parietal, temporal, and frontal cortex, and in the cerebellum. Within the PD group, we found positive correlations between GM volume and cognitive performance for (i) parahippocampal gyrus and occipital lobe and verbal memory, (ii) medial temporal lobe and putamen and visuospatial memory, (iii) middle temporal gyrus and frontal lobe and verbal fluency, and (iv) inferior parietal lobe and cognitive flexibility. These VBM results suggest that in addition to the diffuse structural changes that affect the PD population in general, between-patient differences in regional GM volume may play a role in cognitive heterogeneity.

Despite the advantages of this voxel-based technique, VBM suffers from a major drawback: it does not distinguish between different cortical morphological properties [5]. GM volume is the product of cortical thickness (CTh) and surface area (SA) [6]. There is evidence to suggest that CTh and SA are differentially affected in normal aging [7] and Alzheimer’s disease [8]. Similarly, recent studies suggest that a separate consideration of these two components of GM volume may also be more informative in the context of PD [9–11]. We therefore employed FreeSurfer, a surface-based technique, to measure CTh, SA, and (sub)cortical GM volume in the PD and HC groups originally analyzed with VBM [4]. This approach provided the opportunity to i) investigate specific structural changes related to PD, ii) study the contribution of different aspects of brain structure to cognitive heterogeneity in PD, and iii) compare the use of two common neuroimaging techniques for structural analyses in an identical dataset. We hypothesized to find structural decreases in PD patients when compared with controls, which could be (partly) explained by differences in CTh and SA. Similarly, we expected to find correlations between task performance and structural measures in brain areas that would (partly) overlap with those found in our previous VBM study within the PD sample. Although VBM and FreeSurfer are complementary (i.e. they do not measure the same (sub)cortical characteristics) we expected to replicate the most robust cortical and subcortical effects we found in our previous study.

## Material and Methods

### Participants

A detailed description of the selection procedure of our participants is provided in Gerrits et al (2013). Briefly, we selected 93 idiopathic PD patients from a large, well-documented cohort of the outpatient clinic for movement disorders at the VU University medical center (VUmc), as well as 46 demographically age- and sex-matched HC. Magnetic resonance imaging (MRI) scans and demographic information, such as age and sex, were collected for the entire sample. Due to incorrect cortical reconstruction, we excluded one control participant, resulting in a sample that is almost, but not entirely, identical to the sample used in the VBM study [4]. Within the PD group, we evaluated education level using a scaled Dutch classification system ranging from 1 (did not finish primary school) to 7 (university degree) [12]. We assessed severity of motor symptoms and stage of illness with the motor subscore of the Unified Parkinson’s Disease Rating Scale (UPDRS-III) and Hoehn & Yahr scales [13], respectively. Disease duration was defined as the subjective time interval between the first reported classical motor symptoms and the moment of clinical assessment. We evaluated mood and anxiety symptoms with the Beck Depression Inventory (BDI) [14] and the Beck Anxiety Inventory (BAI) [15], respectively. The cognitive status of our PD cohort was assessed by trained neuropsychologists as part of the standard diagnostic procedure. Of the 93 PD patients, 75 patients did not show evident cognitive impairments, eight patients fulfilled the criteria for mild cognitive impairment and four patients were diagnosed with PD dementia. Six patients could not be classified. All participants gave written informed consent according to the declaration of Helsinki to the protocol, which was approved by the local ethics committee of the VUmc. To summarize, we used the structural scans of 93 patients and 46 healthy participants in our previous VBM study, and used the same scans of all 93 patients, and 45 (out of 46) healthy participants in our current study.

### Neuropsychological assessment

Neuropsychological data were available only for the PD group, and not all patients participated in each cognitive task (see table 1). To evaluate global cognitive status, we used the mini-mental status examination (MMSE) [16, 17]. We assessed verbal memory with the Dutch version of the Rey auditory verbal learning task (RAVLT) and measured both the total number of immediately recalled items after five presentations and the number of items retrieved after a delay [18]. The delayed recall condition of the Rey-Osterrieth complex figure test (ROCFT) was used to evaluate visuospatial memory [19]. We administered the Category fluency task (naming as many animals as possible in 60 seconds) to examine semantic fluency and the Letter fluency task (naming as many words possible starting with D, A and T in 3 trials of 60 seconds each) to assess phonemic verbal fluency. We examined executive functioning with the Stroop color word test [20] and the Trail making test [21]. Interference susceptibility was measured as the time needed for card III of the Stroop Color-Word Test minus the average completion time of Card I (speed of word reading) and II (speed of color naming). We subtracted the completion time on TMT-A from the completion time of TMT-B (TMTB-A) to obtain a measure of cognitive flexibility. The procedures for neuropsychological assessment followed those described by Lezak and colleagues [22].

**Table 1:** Demographic and clinical features of the PD and HC group, and PD subgroups for each neuropsychological test.

	PD total	RAVLT	ROCFT	Stroop	TMTB-A	Category Fluency	Letter Fluency	HC	p-value (PD total vs HC)
Number of participants	93	88	83	86	79	85	80	45	-
tGM	648 ± 66	648 ± 67	651 ± 67	648 ± 66	649 ± 67	650 ± 68	650 ± 65	652 ± 59	-
Sex (male) (%)	61 (65.6)	56 (63.6)	53 (63.9)	54 (62.8)	48 (60.8)	56 (65.9)	52 (65.0)	27 (60.0)	0.52^b^
Age (years) (range)	62.8 ± 10.3(27–88)	62.5 ± 10.2(27–88)	62.2 ± 10.4(27–88)	62.5 ± 10.3(27–88)	62.2 ± 9.9(27–88)	62.5 ± 10.5(27–88)	62.0 ± 10.1(27–84)	60.6 ± 7.8(47–77)	0.19^a^
Education (Verhage)	5 (1–7)	5 (1–7)	5 (1–7)	5 (1–7)	5 (1–7)	5 (1–7)	5 (1–7)	-	-
UPDRS-III score	25.5 ± 10.3	24.2 ± 9.6	23.4 ± 9.2	24.0 ± 9.6	24.1 ± 9.7	24.7 ± 9.9	23.9 ± 9.6	-	-
Hoehn and Yahrstage (range)	2 (1–4)	2 (1–3)	2 (1–3)	2 (1–3)	2 (1–3)	2 (1–4)	2 (1–3)	-	-
Disease duration (years)	3.0 ± 3.2	2.8 ± 2.7	2.8 ± 2.7	2.8 ± 2.7	2.7 ± 2.7	3.0 ± 3.2	2.7 ± 2.6	-	-
DRT (n) (%)	32 (34.4)	30 (34.1)	27 (32.5)	29 (33.7)	28 (35.4)	27 (31.8)	26 (32.5)	-	-
LEDD (mg/day)	509 (100–1590)	492 (100–1590)	522 (150–1590)	495 (100–1590)	509 (150–1590)	525 (100–1590)	490 (100–1590)	-	-
MMSE	27.7 ± 3.0	27.8 ± 2.7	27.7 ± 3.0	28.0 ± 2.1	28.0 ± 2.1	28.0 ± 2.2	28.1 ± 2.1	-	-
BDI	8 (0–32)	8 (0–28)	8 (0–28)	8 (0–28)	8 (0–28)	8 (0–26)	8 (0–28)	-	-
BAI	10 (0–45)	10 (0–45)	10 (0–45)	10 (0–45)	11 (1–45)	10 (0–45)	10 (0–45)	-	-

Data represent mean ± SD or median (range).

Abbreviations: *PD* Parkinson's disease patients; *HC* healthy controls; *RAVLT* Rey Auditory Verbal Learning Test immediate and delayed recall; *ROCFT* Rey Osterrieth Complex Figure Test delayed recall; *Stroop* Stroop word color task; *TMTB-A* Trail Making Test B-A; *tGM* total grey matter volume; *UPDRS-III* Unified Parkinson's Disease Rating Scale-III; *DRT* dopamine replacement therapy; *LEDD* Levodopa equivalent daily dose, computed in the group of medicated patients only; *MMSE* Mini-Mental State Examination; *BDI* Beck Depression Inventory; *BAI* Beck Anxiety Inventory

^a^ Student t test

^b^ Chi squared test

### MRI acquisition and preprocessing

High-resolution structural MRI scans were obtained at the VUmc, using a GE Signa HDxt 3.0-Tesla MRI-scanner (General Electric, Milwaukee, Wisconsin, USA) with an 8-channel head coil. We acquired structural MRI data using a sagittal 3-dimensional gradient-echo T1-weighted sequence (256 x 256 matrix; field of view = 25cm; slice thickness = 1mm; voxel size = 1 x 0.98 x 0.98 mm; TR = 7.8 ms; TE = 3.0 ms; view angle = 12°). Image analysis was carried out with the stable version (v.5.3.0) of the FreeSurfer software (http://surfer.nmr.mgh.harvard.edu) [23–25]. In short, the procedure included: motion correction, intensity normalization, Talairach registration, skull stripping, segmentation of subcortical white matter, tessellation of the GM/white matter (WM) boundary, automated topology correction, and surface deformation. We used a 10 mm (full-width at half-maximum) Gaussian kernel to smooth maps. Finally, FreeSurfer created a surface 3D model of the cortex using intensity and continuity information.

### Cortical analysis

We visually checked the cortical reconstruction of each subject for inaccuracies and manually corrected major topological inaccuracies with vertex edits or control points and subsequently repeated the processing. CTh was calculated as the shortest distance between the GM/WM boundary and pial surface at each vertex across the cortical mantle, measured in millimeters (mm). In addition to vertex-based reconstruction, FreeSurfer automatically parcellated the cortex into 34 gyral-based regions-of-interest (ROIs) per hemisphere, according to the Desikan-Killiany atlas. For each of the 68 cortical parcellations, FreeSurfer calculates i) the average CTh (in mm), ii) total cortical SA of the pial (in mm^2^), and iii) the cortical GM volume (in mm^3^).

### Subcortical analysis

Subcortical volumes were calculated with FreeSurfer’s automated procedure for volumetric measures. Each voxel in the normalized brain volume was assigned to one of 40 labels, using a probabilistic atlas obtained from a manually labeled training set [26]. The labels we used for further analysis were the putamen, caudate nucleus, globus pallidus, nucleus accumbens, brainstem, thalamus, amygdala, hippocampus, ventral diencephalon and the ventricular system. In contrast to our VBM study, the cerebellum was excluded and volumetric measures of the ventricles were included. Last, a measure of total GM (tGM) (in mm^3^) was also computed, consisting of both surface-based cortical GM volume calculations and subcortical voxel counts.

### Statistical analyses

To assess differences in demographic variables between the PD and HC group and PD subgroups for each task, we performed t-tests (for continuous data) and chi-square tests (for categorical data). We checked assumptions of normality and homogeneity of variance with the Shapiro-Wilk test and Levene’s test, respectively. To correct for non-normal distribution, all values of ventricle volume, the TMTB-A score, and Stroop color word interference test scores were log-transformed. We used t-tests and Pearson correlations since parametric assumptions were met for 74% of the data.

### Group differences

A number of statistical tests was performed to assess between-group differences in structural measures. First, we performed a vertex-wise analysis of differences in CTh in FreeSurfer’s statistical program QDEC 1.5, using Monte Carlo-simulations with 10.000 iterations to correct for multiple comparisons and a cluster-wise *p*-value of .05 to display results. Second, surface (i.e. SA per parcellation) and volumetric analyses (i.e. sub-cortical volume estimates calculated by FreeSurfer, and the manually calculated volume estimate per cortical parcellation) were performed in SPSS 20.0 (SPSS, Chicago, IL, USA). For SA and cortical volume, we performed independent t-tests using the 68 parcellations (34 per hemisphere) as dependent variables, group as between-subject factor, and tGM volume as a nuisance variable [8]. Between-group differences in subcortical volume were investigated with the volume of the 23 automatically segmented subcortical regions as dependent variable, group as between-subject factor, and tGM as a nuisance variable. We applied a Bonferroni correction by dividing our *p*-value by the number of cortical areas per hemisphere (*p* < (.05/34) = ~.001) and by the number of sub-cortical structures per hemisphere (*p* < (.05/13) = ~.004) in order to correct for multiple comparisons.

### Correlations with cognitive performance

Since neuropsychological data were only available for the PD patients, correlations between cognitive performance and structural measurements were restricted to this group. We used a GLM model in QDEC 1.5 to correlate CTh at each vertex with scores on the six neuropsychological tasks, while including age, sex, and education as covariates, and applying Monte Carlo-simulations to correct for multiple comparisons. We used a ‘different-onset-same-slope’ model, which assumes that no sex*age interaction exists. For SA, cortical GM, and sub-cortical volume, we computed partial correlations in SPSS 20.0 using each segmentation/parcellation as criterion, neuropsychological test score as predictor, and age, sex, education, and tGM level as covariates. Again, Monte Carlo-simulations (in QDEC) and Bonferroni corrections (in SPSS) were applied to correct for the multiple comparisons.

## Results

The PD and HC group were matched for age (*p* = .19) and sex (*p* = .52). In addition, the PD subgroups for each task were similar regarding education, disease-related variables (i.e. UPDRS III score, Hoehn and Yahr stage, disease duration, dopamine replacement therapy), global cognitive functioning and measures of mood (i.e. depression and anxiety level) (see table 1). On average, patients had a UPDRS III score of 24, a Hoehn and Yahr stage of 2 and a median disease duration of 3 years. The majority of the PD group was still unmedicated at the time of scanning (i.e. only 34% received dopamine replacement therapy).

### Group differences

**Fig 1:**
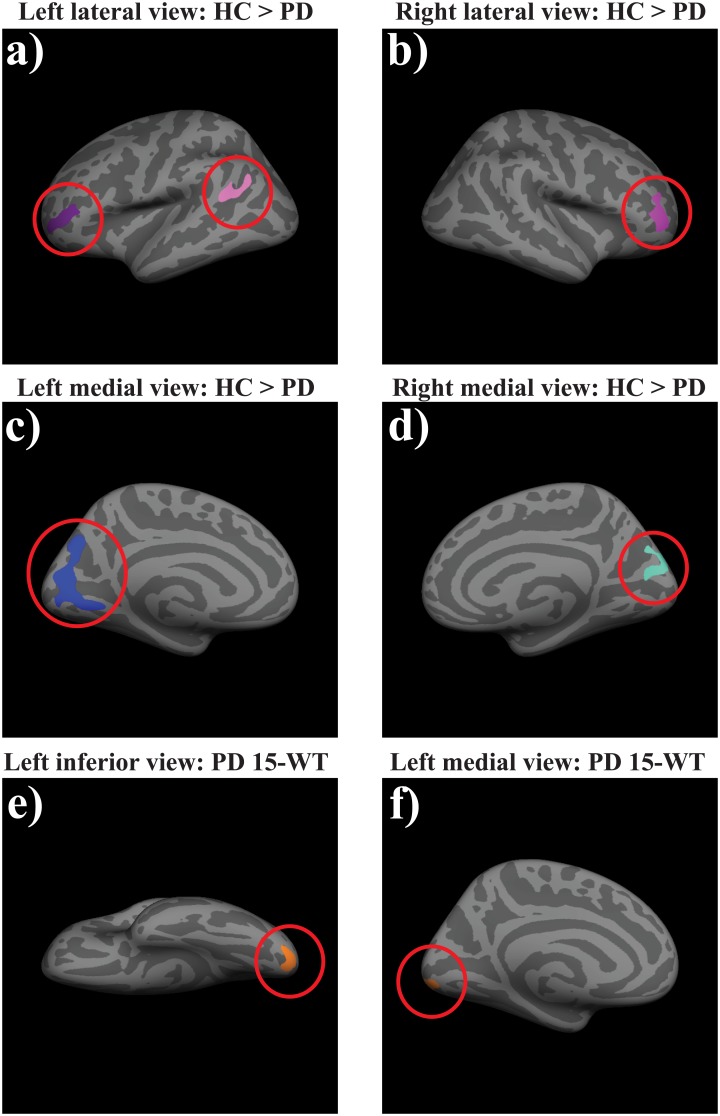
Between-group differences in cortical thickness and thickness and correlation with task performance. HC had increased cortical thickness in the left pericalcarine gyrus, extending to cuneus, precuneus and lingual areas left inferior parietal cortex, bilateral rostral middle frontal cortex, and right cuneus, when compared with PD patients (a-d). Within the PD sample, we found a negative correlation between the left lateral occipital and lingual gyrus and performance on the RAVLT (e-f). Clusters were significant after multiple comparison correction with Monte Carlo simulations.

The vertex-wise CTh analysis showed cortical thinning in PD patients compared with HC in the left pericalcarine gyrus, extending to the cuneus, precuneus and lingual areas, in the left inferior parietal cortex, bilateral rostral middle frontal cortex, and right cuneus (see table 2 and Fig 1a–1d). In addition, PD patients showed enlargement of the third, and bilateral lateral ventricles and left inferior lateral ventricle when compared with HC. For SA, we found that the PD patients had increased cortical SA of the pars triangularis in the right hemisphere (see table 3). No group differences in cortical GM volume were found.

**Table 2:** Vertex-wise cortical thickness group analysis.

Region	Cluster size (mm^2^)	PD	HC				CWP
				X	Y	Z	
L pericalcarine gyrus	1802	1.87 ± 0.13	2.00 ± 0.13	-5	-75	12	< .001
R rostral middle frontal	633	2.24 ± 0.12	2.36 ± 0.14	-40	-49	4	.001
R cuneus	597	1.92 ± 0.13	2.03 ± 0.12	-7	-86	27	.002
L rostral middle frontal	588	2.16 ± 0.13	2.28 ± 0.15	-38	-43	3	.002
L inferior parietal	419	2.37 ± 0.16	2.51 ± 0.15	-38	-62	27	.02

Data represent mean thickness in mm ± SD. Only effects with significant clusterwise-values after Monte Carlo simulations are presented. Coordinates are depicted as peak-coordinates within the MNI305 reference frame

Abbreviations: *PD* Parkinson’s disease patients; *HC* healthy controls; *CWP* clusterwise corecte *p*-value

**Table 3:** (Sub)cortical volume + cortical pial surface area group analysis.

Measurement	Region	PD	HC	*t*	*p*
Subcortical volume (mm^3^) ^a^	3rd ventricle	3.20 ± 0.17	3.09 ± 0.17	14.64	< .001
	L lateral ventricle	4.15 ± 0.23	4.00 ± 0.22	14.35	< .001
	R lateral ventricle	4.11 ± 0.23	3.97 ± 0.23	11.12	.001
	L inferior lateral ventricle	2.71 ± 0.31	2.54 ± 0.27	9.43	.003
Cortical surface area (mm^2^)	R pars triangularis	1875 ± 336	1726 ± 298	10.57	.001

Data represent mean ± SD. Only effects with significant *p*-values after Bonferroni correction are presented.

Abbreviations: *PD* Parkinson’s disease patients; *HC* healthy controls

^a^ All measurements of ventricle volume are log transformed.

### Correlations with cognitive performance

Vertex-wise analysis revealed a negative correlation between CTh in left lateral occipital and lingual areas and performance on the RAVLT immediate recall condition (see Fig 1e and 1f). The volume of the right thalamus showed a negative correlation with performance on the Letter and Category fluency task. The volume and SA of the left pars opercularis correlated negatively with performance on the ROCFT (see table 4).

**Table 4:** Partial correlations of (sub)cortical volume and surface area with neuropsychological task performance, corrected for age, sex, education, and tGM volume.

Measurement	Region	Task	*r*				CWP
				X	Y	Z	
Cortical thickness (mm) ^a^	L lateral occipital and lingual gyrus	RAVLT immediate recall	-.420	-19	-96	-15	.006
Subcortical volume (mm^3^)^b^	R thalamus	Letter Fluency	-.338	-	-	-	.003
	R thalamus	Category Fluency	-.322	-	-	-	.003
Cortical volume (mm^3^)^b^	L pars opercularis	ROCFT	-.374	-	-	-	.001
Surface area (mm^2^) ^b^	L pars opercularis	ROCFT	-.415	-	-	-	< .001

Coordinates are depicted as peak-coordinates within the MNI305 reference frame

Abbreviations: *RAVLT* Rey Auditory Verbal Learning Task; *ROCFT* Rey Osterrieth Complex Figure Test; *Stroop* Stroop word color test; *r* Pearson’s correlation coefficient; *CWP* clusterwise corrected *p*-vale;

^a^ Based on vertex-wise analysis

^b^ Based on parcellation-wise analysis

## Discussion

In this study, we used a surface-based analysis method to investigate structural brain changes in PD and the role of distinct morphological properties on cognitive heterogeneity among patients. Compared with controls, PD patients showed cortical thinning in the right cuneus, left lateral occipital areas, left inferior parietal cortex and the bilateral rostral middle frontal cortex, ventricular enlargement, and increased cortical surface area in the right pars triangularis. Within-group variance in volume of the thalamus, CTh of the left lateral occipital and lingual areas, and cortical volume and SA of the left pars opercularis related to heterogeneity fluency, verbal memory, and visuospatial memory, respectively. As in our VBM study, brain areas showing group differences in morphological properties did not overlap with brain areas in which structural changes were related to cognitive performance. Thus, while PD patients as a group showed atrophy in various regions compared with the HC, cognitive heterogeneity among patients was associated with between-patient structural differences in other regions. These differences may reflect subtle PD-related structural changes that affect only a subgroup of patients. Alternatively, they represent premorbid differences that may have caused some patients to be less vulnerable than others to cognitive impairment as a consequence of the PD-related structural changes observed at a group level.

Various structural imaging studies have consistently shown a negative correlation between structural brain measures (i.e. cortical thickness and / or GM volume), and cognition in PD [27–31]. PD patients with MCI, for example, have faster rates of cortical thinning when compared with patients without MCI [32]. Furthermore, non-demented PD patients who developed PDD within two years after baseline assessment showed a faster rate of cortical thinning than those who did not develop PDD [33]. These two studies further corroborate the relation between brain structure and cognition in a longitudinal design. Within groups of PD patients, correlations have been found between task performance on neuropsychological tests and GM structure, thereby also strengthening the relation between brain structure and cognition [34]. Our study also found associations between cortical thickness / cortical surface area / GM volume and task performance on several neuropsychological tests. Furthermore, we found relatively small areas of reduced cortical thickness in our cohort of patients, which corresponds with findings from other investigations in groups of cognitively preserved PD patients [35–37], although it is important to emphasize that our cohort was not selected to represent a unitary cognitive status (e.g. not cognitively impaired / cognitively impaired).

Despite a lack of overall consensus, neuro-pathological studies have suggested that differences in cortical thickness and GM volume primarily represent differences in neuronal structural complexity (i.e. synapses and dendritic arborisation) and not neurons *per se*, although the influence of (mircro)glia, blood vessels and, neuronal size cannot be fully excluded [38]. This hypothesis concurs with other longitudinal observations in which GM volume [39, 40] and cortical thickness [41] increased after training in task-related areas and further supports the relation between structure and function [42]. Relating this hypothesis to our current findings suggests that an optimal structural complexity (i.e. synaptic efficiency) in certain areas leads to an increased task performance on some neuropsychological tasks.

Although we used the same dataset in the current study as in our previous VBM analysis [4], there was surprisingly little overlap between the studies in the areas in which we found significant effects. Since our study was not designed to specifically investigate between-technique differences, we will only shortly discuss a number of possible explanations why the current findings deviate from our previous results. i) In our VBM study, we applied an uncorrected *p*-value of .001 with an extent threshold of 50 voxels, whereas the present study employs Monte Carlo simulations and Bonferroni corrections, which are statistically more stringent [43]. To exclude the effects of potential false-positive findings, we reanalyzed our data using both techniques while employing an FDR correction. For the VBM analysis, no effects survived the statistical threshold, whereas in the FreeSurfer analyses we found clusters of decreased cortical thickness in the left parietal, occipital and frontal areas when comparing patients with controls. So also when employing a similar statistical threshold, the results between our two studies still differ. ii) Whereas FreeSurfer calculates the total volume of a cortical parcellation or subcortical segmentation, VBM assesses GM volume on a voxel-by-voxel basis. VBM might, therefore, be more sensitive to detect small local effects that may be ‘averaged out’ when measured over a larger area. However, volume-based techniques, such as VBM, are prone to partial volume effects, which might lead to erroneous segmentation and registration, and thereby to an overestimation of GM differences [44, 45]. Also minor methodological variations, such as different spatial transformations or smoothing procedures can alter results in a way similar to the biologic differences under investigation [46]. Since FreeSurfer is a surface-based technique, and thereby differentially affected by these important preprocessing steps, this further hinders the between-technique comparison. iii) Cortical GM volume as a measure of brain structure is different from CTh and SA. Our FreeSurfer results show, in accordance with earlier studies [9–11], that CTh and SA are differentially affected in PD. Since the product of their combined influence is not uniform across the cortex, cortical GM volume may not show overlap with either measure, or effects (e.g. increased SA / decreased CTh) in opposite directions may cancel each other out. iv) This cohort of patients was, overall, still in an early disease stage, and cognitively relatively well-preserved. Several other studies have investigated structural changes in early stage PD, and found little or no atrophy in cognitively preserved cohorts, comparable to ours [35, 37]. The areas in which atrophy was described varied considerably between studies, thus suggesting that the atrophy is subtle and topographically non-specific, in contrast with, for example, hippocampal atrophy in Alzheimer’s disease. We argue that if the structural differences had been more pronounced, both techniques would have detected them. Our results confirm previous studies by showing that there is indeed atrophy in relatively early stage PD, but that it is, if anything, subtle and spread over various brain areas. Also the enlargement of the third and lateral ventricles indicates a diffuse and non-specific degenerative process.

Several results are consistent with previous data obtained using FreeSurfer in PD, mainly concerning CTh reductions in the bilateral rostral middle frontal cortex, bilateral cuneus and left inferior parietal areas [47–49], as well as the enlargement of the third and lateral ventricles [50, 51]. Also the positive correlation between SA of the left medial orbitofrontal cortex and Stroop task performance is in accordance with the involvement of this area in response inhibition [52]. In contrast, the negative correlation between verbal memory performance and CTh of the lateral occipital and lingual cortex is not in line with earlier findings. Pellicano et al. [53] reported a positive correlation between verbal memory performance and thickness in occipital areas (i.e. the fusiform area) in PD. Also the negative correlations between the left pars opercularis and the right thalamus with visuospatial memory and verbal fluency, respectively, are difficult to interpret, although numerous cognitive processes have been associated with these areas [54] [55–57]. Future studies should replicate these findings before any definite statements can be made. In addition, since both VBM [58] and FreeSurfer [59] have problems segmenting the thalamus from the surrounding WM, we advise caution when interpreting the correlation between the thalamus and verbal fluency we found in the current, but not the previous, (VBM) study, since these conflicting results could indicate a spurious finding. Replication in future research is therefore warranted.

One could speculate that the negative correlations can be interpreted as a form of pruning to get a more efficient organisation, and thus less thickness equals more efficiency, thereby leading to a better task performance. This, however, is not in line with findings in which an increase in thickness is found after (cognitive) training in task-related areas (see e.g. [41]. Future studies should therefore replicate these negative correlations findings before any definite statements can be made.

To our knowledge, this is the first study that compared VBM and FreeSurfer data in the same cohort of PD patients to study the relation between brain structure and cognitive performance. Strengths of our study include our relatively large and well-powered [60] sample and the fact that we controlled for various confounding factors such as age, sex and education. An important limitation, however, is the absence of neuropsychological test scores from HC. Conclusions based on the correlations between brain structure and cognitive performance should therefore be interpreted with caution, as they may not be specific to PD. Furthermore, FreeSurfer, by default, calculates cortical thickness as the shortest (Euclidian) distance between two nearest vertices; once from the pial surface to the GM/WM boundary, and once from the GM/WM boundary to the pial surface. These two values are then averaged to produce a thickness value at that node. Although there is no golden standard or general consensus as to which measure is best [61, 62], it is important to keep in mind that shortest distance is not the only way to calculate cortical thickness. Some have argued that other measures, such as linked-distance, might be more sensitive to differences in thickness (see e.g. [63]).

Future research should include a longitudinal approach to gain more insight into how structural changes relate to cognitive status over time. It would also be insightful to include patients with a more diverse cognitive profile to make the sample more heterogeneous, or subdivide the sample into subgroups based on cognitive status (e.g. cognitively not impaired; cognitively impaired; demented).

## Conclusions

The results of the current study suggest that PD is associated with cortical thinning and ventricular enlargement, and that cognitive heterogeneity within the PD population is associated with subtle differences in CTh, SA, and (sub)cortical GM volume. Our results obtained with FreeSurfer support the hypothesis that CTh and SA are differentially affected by the disease, and have diverse associations with cognition. This underlines the necessity to take distinct morphological properties of brain areas into account in the context of PD. By comparing GM volume effects obtained with FreeSurfer and VBM, we have provided evidence that their methodological and technical differences can yield non-overlapping results in the same cohort of participants. We think researchers should be aware of the consequences of the choice of technique on their results, and we recommend that future research should further investigate why two structure-based analysis techniques yield different findings.
